# Exploring Cluster-Dependent Antibacterial Activities and Resistance Pathways of NOSO-502 and Colistin against Enterobacter cloacae Complex Species

**DOI:** 10.1128/aac.00776-22

**Published:** 2022-10-06

**Authors:** Lucile Pantel, François Guérin, Marine Serri, François Gravey, Jessica Houard, Kelly Maurent, Marie Attwood, Alan Noel, Alasdair MacGowan, Emilie Racine, Vincent Cattoir, Maxime Gualtieri

**Affiliations:** a Nosopharm, Nîmes, France; b CHU de Rennes, Service de Bactériologie-Hygiène Hospitalière, Rennes, France; c Université de Rennes 1, Unité INSERM U1230 BRM, Rennes, France; d Université de Caen Normandie, Dynamicure, INSERM U1311, CHU de Caen, Caen, France; e CHU de Caen, service de bactériologie, Caen, France; f Bristol Centre for Antimicrobial Research and Evaluation (BCARE), Infection Sciences, Southmead Hospital, Bristol, United Kingdom

**Keywords:** NOSO-502, colistin, *Enterobacter cloacae* complex, mechanism of resistance, KexD efflux pump, hetero-resistance, CrrAB two-component-system, PhoPQ

## Abstract

The Enterobacter cloacae complex (ECC) is a group of diverse environmental and clinically relevant bacterial species associated with a variety of infections in humans. ECC have emerged as one of the leading causes of nosocomial infections worldwide. The purpose of this paper is to evaluate the activity of NOSO-502 and colistin (CST) against a panel of ECC clinical isolates, including different Hoffmann’s clusters strains, and to investigate the associated resistance mechanisms. NOSO-502 is the first preclinical candidate of a novel antibiotic class, the odilorhabdins (ODLs). MIC_50_ and MIC_90_ of NOSO-502 against ECC are 1 μg/mL and 2 μg/mL, respectively, with a MIC range from 0.5 μg/mL to 32 μg/mL. Only strains belonging to clusters XI and XII showed decreased susceptibility to both NOSO-502 and CST while isolates from clusters I, II, IV, and IX were only resistant to CST. To understand this phenomenon, E. cloacae ATCC 13047 from cluster XI was chosen for further study. Results revealed that the two-component system ECL_01761-ECL_01762 (ortholog of CrrAB from Klebsiella pneumoniae) induces NOSO-502 hetero-resistance by expression regulation of the ECL_01758 efflux pump component (ortholog of KexD from K. pneumoniae) which could compete with AcrB to work with the multidrug efflux pump proteins AcrA and TolC. In E. cloacae ATCC 13047, CST-hetero-resistance is conferred *via* modification of the lipid A by addition of 4-amino-4-deoxy-l-arabinose controlled by PhoPQ. We identified that the response regulator ECL_01761 is also involved in this resistance pathway by regulating the expression of the ECL_01760 membrane transporter.

## INTRODUCTION

The Enterobacter cloacae complex (ECC) is a group of diverse bacterial species of clinical and environmental relevance (1). These facultative anaerobic bacteria belonging to the Gram-negative *Enterobacteriales* family are widely present in nature and are part of the gut commensal microbiota of animal and human populations (2). ECC species were clustered by Hoffmann and Roggenkamp in 12 groups on the basis of DNA sequence of their hsp60 genes (designated C-I to C-XII, Table S1) (3). Some of them, frequently belonging to C-III, C-VI, and C-VIII, are associated with a variety of human infections and have emerged as one of the leading causes of nosocomial infections worldwide, accounting for up to 5% of hospital-acquired pneumonia and bacteremia, 4% of nosocomial urinary tract infections, and 10% of postsurgical peritonitis (4). The intrinsic resistance of ECC to several antibiotics and their ability to acquire resistance to many others, including the last resort antibiotics such as carbapenems and colistin (CST), makes some infections caused by ECC difficult to treat (5, 6). Indeed, these species are intrinsically resistant to aminopenicillins and first- and second-generation cephalosporins due to the presence of a chromosomal inducible AmpC β-lactamase (7). Moreover, the acquisition of plasmids carrying extended-spectrum β-lactamases (ESBL)-encoding genes confers resistance to most clinically relevant β-lactams (8). Most worrisome, many studies have also reported the global emergence of carbapenem-resistant E. cloacae (CREC), suggesting accelerating resistance acquisition in this organism (9–11). Carbapenem resistance in ECC results from either the constitutive overexpression of AmpC/ESBL combined with decreased permeability or the acquisition of plasmid-encoded carbapenemase genes (e.g., *bla*_KPC_, *bla*_NDM_, or *bla*_OXA-48-like_) (7). In 2013, the need to develop new antibiotics active against carbapenem-resistant *Enterobacterales* (including CREC strains) was classified as urgent by the U.S. Centers for Disease Control and Prevention (CDC) (12). The status of CST is different from that of other antibiotics. This drug is a member of the polymyxin family and was used for the treatment of various Gram-negative infections. Its use by intravenous route was abandoned in the early 1980s because of significant side effects and the introduction into clinical practice of less toxic antibiotics (13). With the increasing prevalence of infections caused by multidrug-resistant (MDR) Gram-negative bacteria and the failure to develop new effective antibiotics, CST has reemerged as therapeutic option for many infections, including those due to ECC (14). Currently, many programs that aim at designing novel polymyxin derivatives are under clinical development (15). Mechanistically, CST is a cyclic cationic lipopeptide that binds to the lipid A part of the LPS, inducing an outer membrane permeabilization and an inner membrane disruption, leading to cell lysis. Consequently, LPS modification by addition of positively charged moieties is the main mechanism of resistance to CST. In *Enterobacterales*, lipid A modification mainly occurs by the addition of phosphoethanolamine (PEtN) or 4-amino-4-deoxy-l-arabinose (L-Ara4N) with regulation by the two-component systems (TCS) PmrAB and/or PhoPQ in response to environmental signals such as the presence of cationic antimicrobial peptides (CAMPs), low magnesium, or acidic pH. PmrAB can activate both *arnBCADTEF* operon expression encoding enzymes responsible for the synthesis and transfer of the L-Ara4N to lipid A but also the pEtN transferase PmrC. Depending on the species, PhoPQ can either directly activate the *arnBCADTEF* operon or indirectly activate by cross-activation of PmrAB via the connector protein PmrD (16). In ECC, CST resistance is regularly associated with a cluster-dependent hetero-resistance phenotype, a phenomenon observed in different Gram-negative and Gram-positive species, in which the major population of susceptible cells is killed in the presence of a given antibiotic whereas a preexisting subpopulation of resistant cells can rapidly multiply (17). It has been postulated that it may cause antibiotic treatment failure and be induced by the host immune system (18, 19). As expected, CST-resistant clinical ECC have also emerged with a high prevalence in some studies (20). In the race against time to develop new antibiotics, the odilorhabdins (ODLs) have emerged as a promising new family. ODL members are cationic peptides that specifically inhibit the bacterial translation by interacting with the 30S subunit of the bacterial ribosome (21, 22). NOSO-502 is the first preclinical candidate of this novel antibiotic class (23). In a recent publication, Racine et al. reported that NOSO-502 exhibits potent activity against MDR colistin-resistant, and carbapenemase-producing *Enterobacterales*, including ECC isolates from different European hospitals (KPC-2, NDM-1, OXA-48) (23). NOSO-502 and CST coresistant K. pneumoniae mutants bearing mutations in *crrB* gene were identified (23). CrrB is a signal-transducing histidine kinase and CrrA is an adjacent response regulator belonging to a TCS named CrrAB. It was established that mutations in the CrrAB TCS induce resistance to NOSO-502 by an upregulation of the efflux pump component KexD when it generates CST resistance by addition of L-Ara4N or pEtN to LPS regulated by the PmrAB or PhoPQ TCS, and via the protein CrrC (24). KexD is predicted to be an energy-dependent efflux pump subunit belonging to the resistance nodulation division (RND) family and CrrC a modulator that interacts with PmrAB to alter *arn* operon expression (25).

The purpose of this study was first to evaluate the activity of NOSO-502 against a large panel of ECC clinical isolates, from different Hoffmann’s clusters, and then to investigate the associated resistance mechanisms. Indeed, understanding and anticipating the emergence of resistance is an essential step in the development of new antibiotics. Our preliminary results confirmed the potent antibacterial activity of NOSO-502 against the most problematic ECC strains, but also surprisingly highlighted two specific clusters were less susceptible to both NOSO-502 and CST, leading us to analyses the associated mechanisms of resistance. A new essential TCS mediating hetero-resistance to both antibiotics by different molecular mechanisms that have never been investigated among ECC members was identified.

## RESULTS

### Antibacterial activity of NOSO-502 against clinical ECC strains is cluster dependent.

To determine whether NOSO-502 antibacterial activity was Hoffmann’s cluster dependent, we determined MIC values of this compound and those of CST against a panel of 25 ECC clinical isolates from several French hospitals belonging to the 12 different clusters (C-I to C-XII) and against the reference strain E. cloacae subsp*. cloacae* ATCC13047 (ECL13047), a C-XI member exhibiting CST resistance with an associated hetero-resistance phenotype (26). NOSO-502 showed potent antibacterial activity against ECC species from C-I to C-X with MIC values between 1 and 2 μg/mL. Only strains belonging to clusters XI and XII displayed decreased susceptibility to NOSO-502 but also to CST (Table 1). Nevertheless, it is important to note that CST-resistant strains from other clusters (C-I, C-II, C-IV, and C-IX) remain susceptible to NOSO-502.

**TABLE 1:** MIC of NOSO-502 and colistin against ECC strains belonging to different clusters

Cluster	Strain	Origin	MIC (μg/mL)	
			NOS	CST
I	ECL_28	Tracheal aspirate	1	>2,048
	CHV23	Pus	1	64
II	ECL_16	Abscess	2	64
	ECL_123	Urine	1	256
	ECL_140	Blood culture	1	32
III	ECL_26	Urine	1	1
	ECL_31	Urine	2	1
IV	ECL_18	Pus	2	32
	ECL_166	Blood culture	2	256
V	ECL_11	Abscess	1	1
	ECL_32	Urine	1	0.5
VI	ECL_25	Catheter	2	1
	ECL_30	Urine	1	1
VII	ECL_121	Pus	1	0.5
	ECL_122	Pus	2	128
VIII	ECL_01	Protected distal bronchial	1	1
	ECL_29	Urine	1	0.5
IX	ECL_146	Pus	2	32
	ECL_JO36	Pus	1	64
X	CHV27	Pus	1	0.5
	CAE15	Pus	1	1
XI	ECL_JO18	Pus	256	256
	ECL_JO24	Pus	128	256
	ECL13047	Reference strain	256	256
XII	ECL_JO20	Pus	8	256
	RMS39	Pus	8	128

MIC values of NOSO-502, CST, and comparators were then determined against a collection of 50 recent ECC clinical isolates collected from several United Kingdom hospitals. MIC_50_ and MIC_90_ of NOSO-502 were 1 μg/mL and 2 μg/mL, respectively (Table 2). Only two isolates of the panel (E. cloacae C1.320, and E. cloacae C1.335) exhibited NOSO-502 MIC values higher than the MIC_90_ (32 μg/mL and 16 μg/mL) (Table S2). These strains were also resistant to CST with MICs >64 μg/mL. Partial sequence analysis of the *hsp60* gene showed that both strains belonged to C-XII. According to the EUCAST guidelines (CST-resistance >2 μg/mL), five other strains, including three *E. bugandensis* (C-IX), and two E. cloacae (C-V), were also resistant to CST but exhibited NOSO-502 MIC values comprised between 1 and 2 μg/mL. Two carbapenemase-producing strains of the panel carrying *bla*_NDM-1_ (C1.330) and *bla*_IMP-4_ (C1.332) displayed NOSO-502 MIC value of 1 μg/mL (Table S2).

**TABLE 2:** MIC_50_, MIC_90_, and MIC ranges of NOSO-502 and antimicrobial agents against a panel of recent ECC clinical isolates from the United Kingdom

Antimicrobial agent	Enterobacter cloacae clinical isolates (*n* = 50)		
	MIC_50_ (μg/mL)	MIC_90_ (μg/mL)	Range (μg/mL)
NOSO-502	1	2	0.5 to 32
Amikacin	1	1	0.5 to >64
Ceftazidime-Avibactam	4	>16	0.12 to >16
Ciprofloxacin	0.015	0.25	0.004 to >1
Colistin	0.5	8	<012 to >64
Meropenem	0.06	0.25	0.015 to >1
Tigecycline	1	2	0.5 to 4

### ECL13047 exhibits hetero-resistance to NOSO-502 and CST.

**FIG 1:**
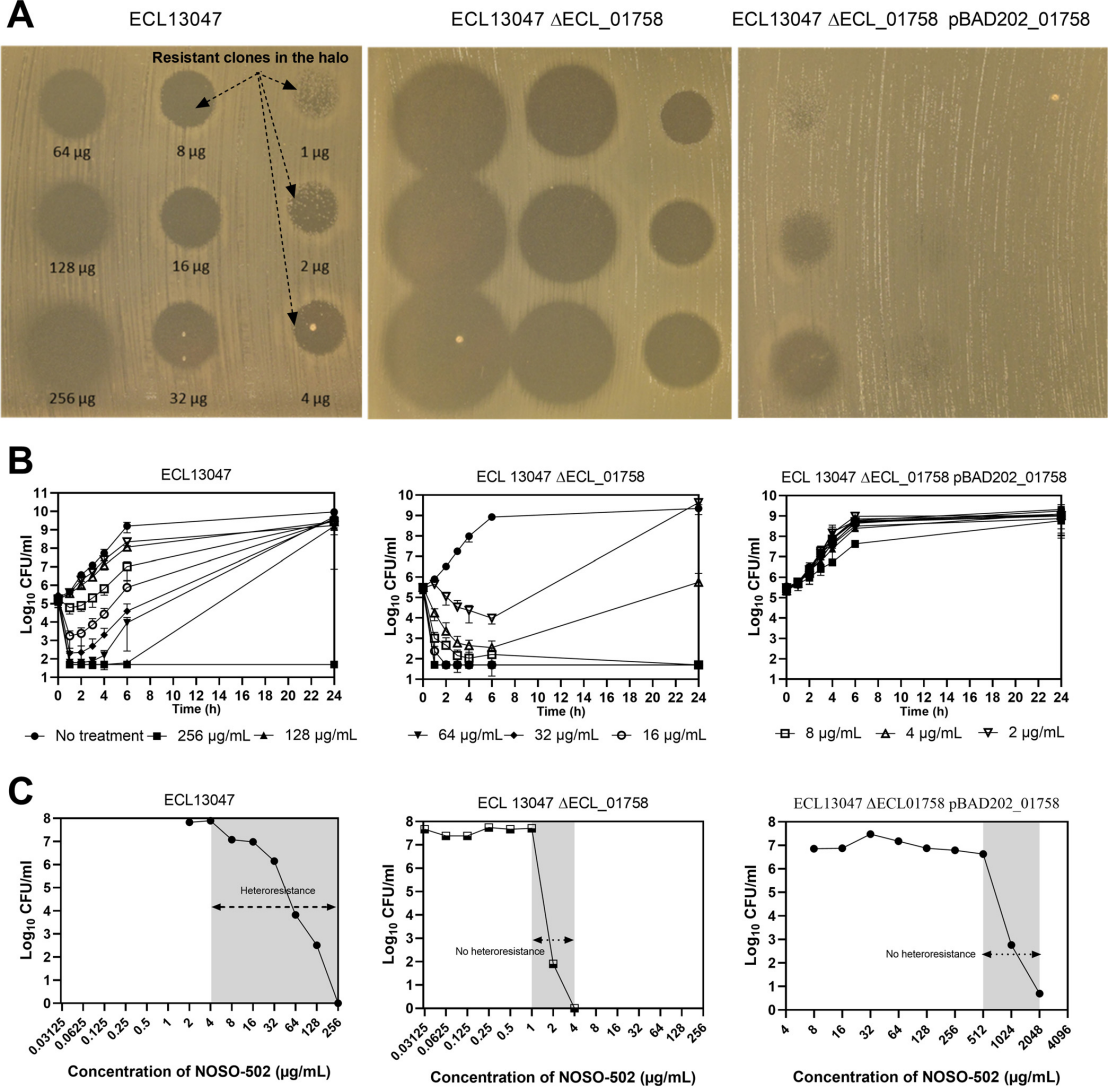
Determination of NOSO-502 hetero-resistance in ECL13047, ECL13047 Δ*ECL_01758*, and ECL13047 Δ*ECL_01758* pBAD202_*01758* strains by (A) agar diffusion assay; (B) time-kill study; (C) population analysis profile (PAP) method.

**FIG 2:**
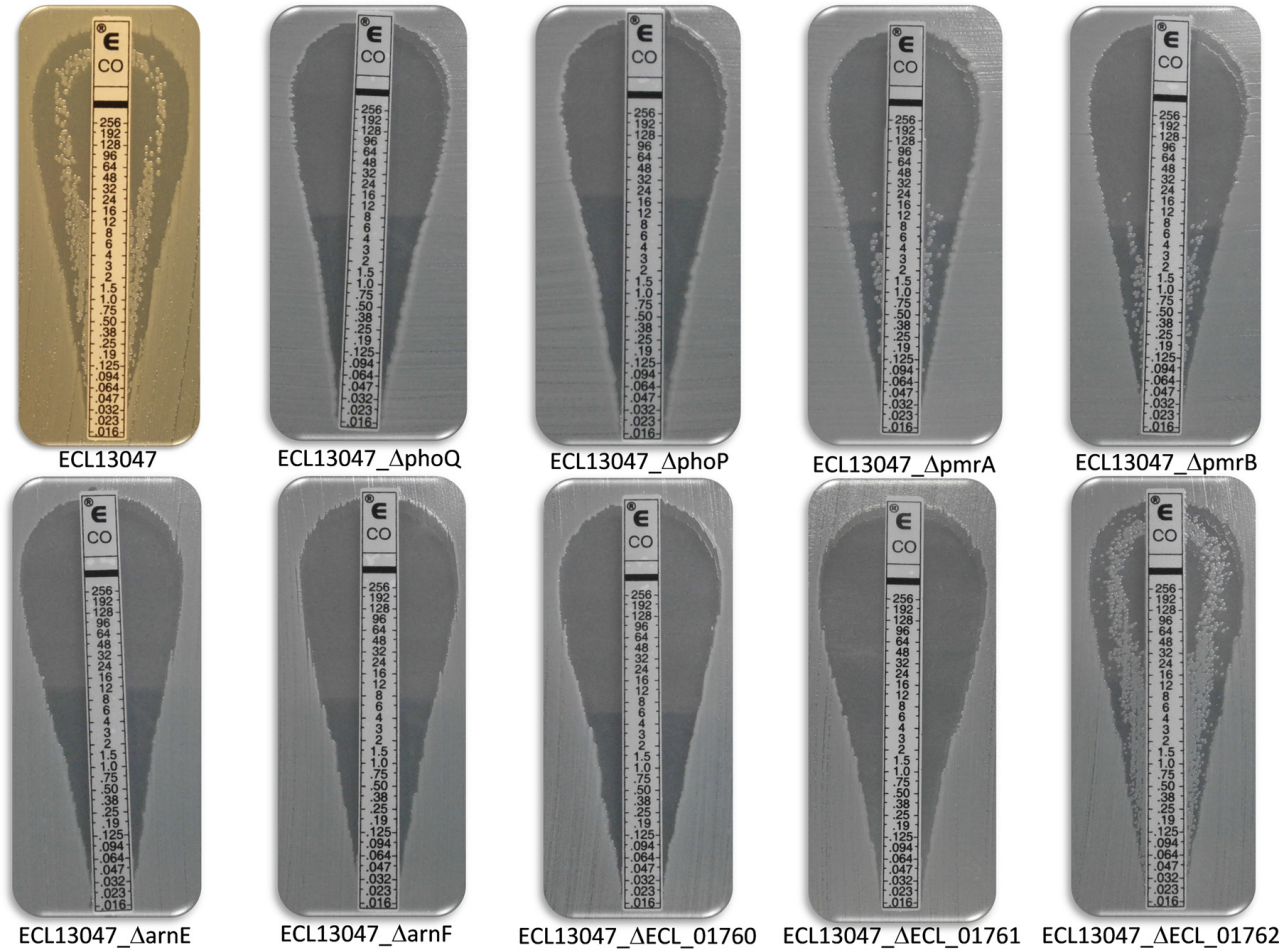
Colistin Etest strips of ECL13047, ECL13047Δ*phoP*, ECL13047Δ*phoQ*, ECL13047Δ*arnE*, ECL13047Δ*arnF*, ECL13047Δ*pmrA*, ECL13047Δ*pmrB*, ECL13047Δ*ECL_01760*, ECL13047Δ*ECL_01761*, and ECL13047Δ*ECL_01762* mutant strains to visualize hetero-resistant colonies.

According to the broth microdilution method (BMD), MIC value of NOSO-502 against ECL13047 was 256 μg/mL (Table 1). Interestingly, in an agar diffusion assay, few colonies were able to grow in the clear zone of inhibition containing a gradient of NOSO-502 quantity (1 μg to 256 μg), demonstrating that most of ECL13047 cells were susceptible to these NOSO-502 concentrations (Fig. 1A). These results were confirmed by the survival curves of ECL13047 with NOSO-502 tested at subinhibitory concentration (between MIC/128 and MIC), in which a rapid bactericidal activity, causing a 3-log decrease in CFU/mL at 1 h, followed by substantial regrowth at all concentrations greater than 16 μg/mL was observed (Fig. 1B). Using the population analysis profile (PAP) method, we determined that the proportion of resistant subpopulations that were able to grow in the presence of 32 μg/mL to 128 μg/mL concentrations of NOSO-502 was on the order of 1.4% to 0.0003%, respectively (Fig. 1C). The reversion phenomenon was then evaluated from NOSO-502-resistant clones selected during PAP study at MIC/8 (32 μg/mL). After culturing in broth medium without antibiotic, similar rate of resistant bacteria at 32 μg/mL was observed for the wild-type strain showing that the NOSO-502 resistance profile of the subpopulation was reversible. Resistant subpopulation has also been identified by different methods in the two other C-XI strains tested (ECL_JO18, ECL_JO24) while no NOSO-502 hetero-resistance was observed in C-XII strains (ECL_JO20, RMS39) (Fig. S2). As previously described, colistin hetero-resistance in ECL13047 was also detected using CST Etest strip on Mueller–Hinton agar plates (Fig. 2).

### ECL13047 displays resistance to NOSO-502 and CST via different pathways.

In ECL13047, lipid A is exclusively modified by addition of L-Ara4N to induce CST hetero-resistance by a mechanism involving the PhoPQ TCS and the *arnBCADTEF* operon (16). To elucidate the underlying mechanisms that regulate resistance to NOSO-502, we analyzed a collection of ECL13047 isogenic strains by determining MIC values using the BMD method against ECL13047 and deletion mutants *ΔphoP*, *ΔphoQ*, *ΔpmrA*, *ΔpmrB*, *ΔpmrAB*, *ΔarnE*, and *ΔarnF*. As previously reported, ECL13047 was resistant to high doses of CST (MIC of 256 μg/mL), whereas all mutants except Δ*pmrA*, and Δ*pmrB* were susceptible to low concentration of CST (MIC between 1 and 4 μg/mL) (Table 3) (16). In contrast, wild type and all deletion mutants exhibited high NOSO-502 MIC values (between 32 μg/mL and 512 μg/mL), showing that the pathways conferring NOSO-502 and CST hetero-resistance in ECL13047 are different (Table 3). The absence of CST-resistant colonies within the clear zone of inhibition was observed using the Etest method with ECL13047 deleted of *phoP*, *phoQ*, *phoPQ*, *arnE*, *arnF*, or *arnBCADTEF* genes while CST-hetero-resistant clones were detected in mutants deleted for *pmrA*, *pmrB*, or *pmrAB* genes (Fig. 2).

**TABLE 3:** MIC of NOSO-502 and CST against Enterobacter cloacae *subsp. cloacae* ATCC13047 strain and deletion mutants

	MIC in μg/mL	
Strain	NOSO-502	Colistin
ECL13047	256	256
ECL13047Δ*pmrA*	256	128
ECL13047Δ*pmrB*	128	128
ECL13047Δ*pmrAB*	256	128
ECL13047Δ*phoP*	256	**2 (128)** ^*a*^
ECL13047Δ*phoQ*	256	**4 (64)**
ECL13047Δ*phoPQ*	256	**2 (128)**
ECL13047Δ*arnE*	**32 (8)**	**1 (256)**
ECL13047Δ*arnF*	512	**1 (256)**
ECL13047Δ*arnBCADTEF*	256	**1 (256)**
ECL13047Δ*ECL_01758*	**4 (64)**	**64 (4)**
ECL13047Δ*ECL_01758* pBAD202ΩECL_01758	**1024 (4)**	512
ECL13047Δ*ECL_01759*	**16 (16)**	128
ECL13047Δ*ECL_01759* pBAD202ΩECL_01759	**16 (16)**	256
ECL13047Δ*ECL_01760*	128	**1 (256)**
ECL13047Δ*ECL_01760* pBAD202ΩECL_01760	128	128
ECL13047Δ*ECL_01761*	**2 (128)**	**1 (256)**
ECL13047Δ*ECL_01761* pBAD202ΩECL_01761	**64 (4)**	**2,048 (8)**
ECL13047Δ*ECL_01762*	**32 (8)**	512
ECL13047Δ*ECL_01762* pBAD202ΩECL_01762	**2 (128)**	256
ECL13047Δ*ECL_01762* ΔphoQ	**8 (32)**	**0.5 (512)**
ECL13047Δ*acrB*	**2048 (8)**	256
ECL13047Δ*acrB* ΔECL_01758	**2 (128)**	**32 (8)**
ECL13047Δ*acrB* pBAD202	**2048 (8)**	128
ECL13047Δ*acrB* pBAD202ΩECL_01758	**>2048 (>8)**	256
ECL13047Δ*acrA*	**8 (32)**	**64 (4)**
ECL13047Δ*tolC*	**2 (128)**	**64 (4)**

a Figures in bold are MICs that are significantly different (fold changes of ≥|4| are indicated in parentheses) from the MICs of the ECL13047 strain.

### ECL_01758, an ortholog of the KexD efflux pump, contributes to NOSO-502 hetero-resistance in ECL13047.

**FIG 3:**
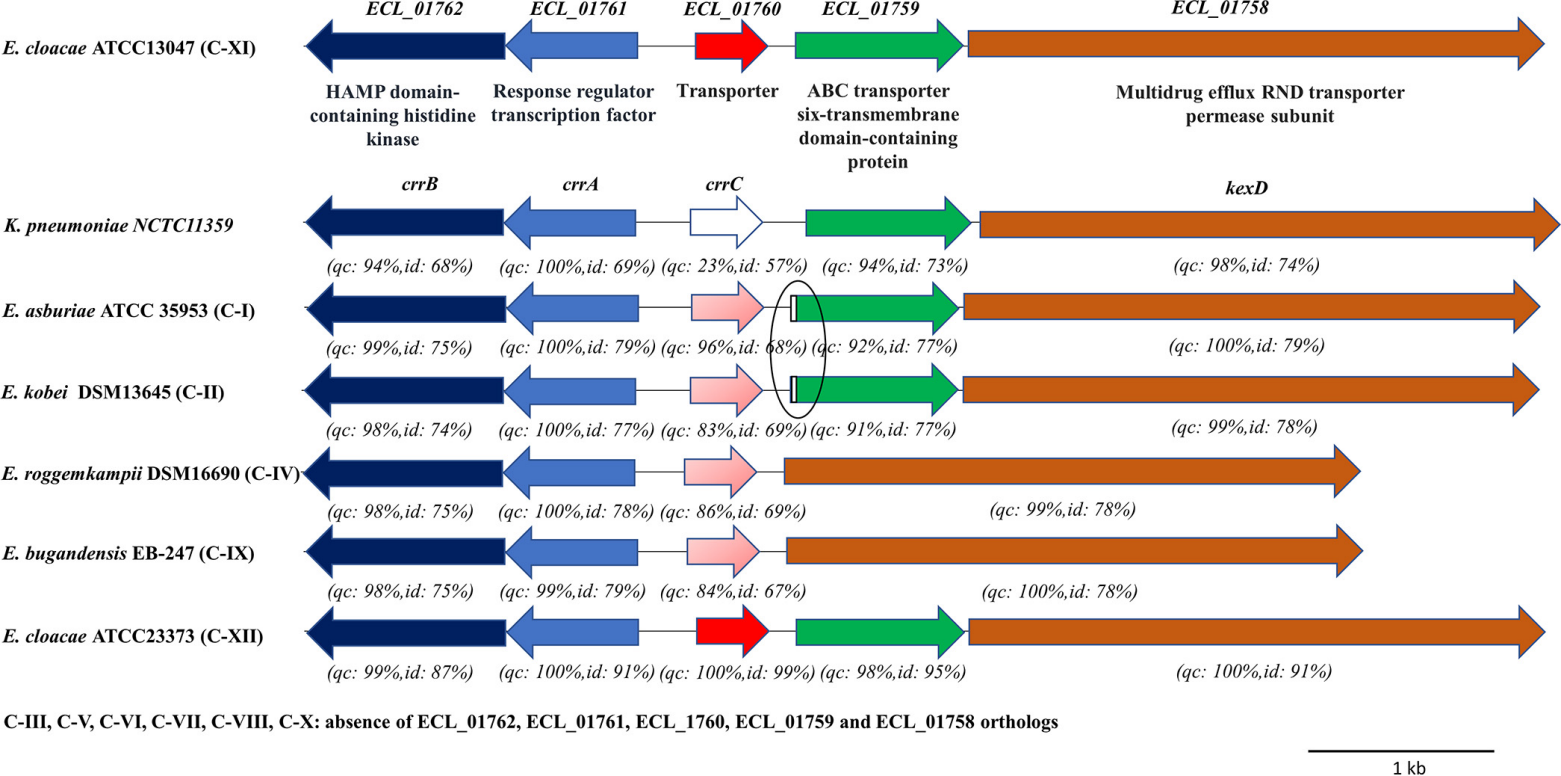
Illustration of the *ECL_01761/ECL_01762* operon organization and its neighboring genes *ECL_01758*, *ECL_01759*, *ECL_01760* in E. cloacae ATCC 13047, K. pneumoniae NCTC11359 and ECC strains from clusters I, II, IV, IX, and XII. Query coverage (qc) and nucleotide sequence identity (id) of strains compared with E. cloacae ATCC 13047 sequences are indicated in parentheses. The small circle corresponds to a low conserved region.

Orthologous genes *ECL_01758*, *ECL_01761*, and *ECL_01762*, displaying 74%, 69%, and 68% nucleotide identity with K. pneumoniae NCTC 11359 *kexD*, *crrA*, and *crrB*, respectively, were identified in ECL13047. Remarkably, organization of the TCS operon and the putative RND-type efflux pump locus was preserved between both strains (Fig. 3).

To define the role of the efflux pump component ECL_01758 in NOSO-502 resistance, deletion of this gene was constructed in the ECL13047 parental strain. Deletion decreased by a 64-fold factor the MIC value of NOSO-502 (MIC = 4 μg/mL) while the wild-type resistant phenotype was restored in the *trans*-complemented strain (MIC = 1,024 μg/mL), confirming that this efflux pump is necessary and sufficient to induce NOSO-502 resistance in ECL13047 (Table 3).

The absence of hetero-resistant subpopulations in agar diffusion assay, PAP analysis, and time-kill study carried out with ECL13047ΔECL_01758 showed that ECL_01758 contributes to NOSO-502 hetero-resistance in ECL13047 (Fig. 1A to C).

### ECL_01758 expression is regulated by the response regulator ECL_01761 and by different histidine kinase, including ECL_01762 and PhoQ.

To assess the extent to which NOSO-502 resistance conferred by ECL_01758 is dependent on the TCS ECL_01761/ECL_01762, mutants with deletion of each gene were generated. Surprisingly, while the *ECL_01761* deletion mutant exhibited substantial loss of resistance to NOSO-502 (MIC = 2 μg/mL), the *ECL_01762* mutant showed only a limited decrease (MIC = 32 μg/mL). Restoration of resistance to NOSO-502 was obtained by *ECL_01761* complementation (MIC= 64 μg/mL) while more remarkably, a total loss of resistance was observed by *ECL_01762* complementation (MIC = 2 μg/mL) (Table 3). These results were confirmed by agar diffusion assays in which a strong increase of the bacterial growth inhibition diameter was observed with the *ECL_01761* or *ECL_01762* deletion mutants compared with ECL13047. While considering this last mutant, resistant colonies were still present at the periphery of the inhibition zone. The *ECL_01762* deletion mutant complemented with *ECL_01762* exhibited similar inhibition diameter profile to its parent but most of peripheric resistant bacteria were eliminated, confirming the role of ECL_01762 in the regulation of resistance to NOSO-502 in ECL 13047 (Fig. S1). These findings are also consistent with the high probability of an interdependent regulation of ECL_01761 by ECL_01762 and by other histidine kinases.

Pantel et al. described the selection of NOSO-502 resistant K. pneumoniae mutants with substitutions on CrrB (ortholog of ECL_01762) from a parental strain exhibiting a complete functional impairment of PhoQ. Interestingly, we obtained a NOSO-502 MIC value of 8 μg/mL against ECL13047 deleted of both histidine kinases ECL_01762 and PhoQ, confirming their potential role in the regulation of ECL_01761 and/or ECL_01758 (Table 3). However, other histidine kinases may be involved in the NOSO-502 resistance pathway in ECL13047.

**FIG 4:**
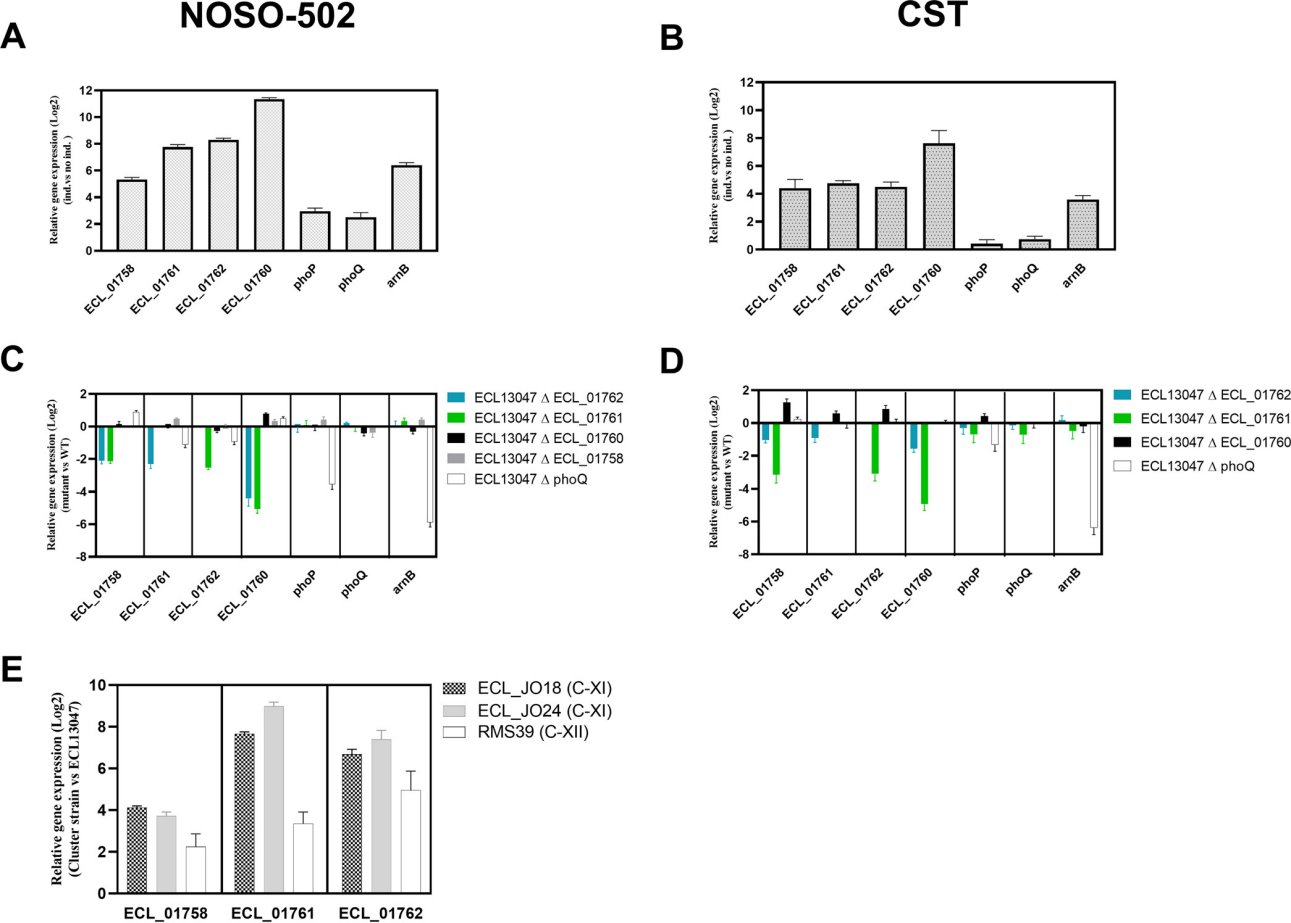
RT-qPCR comparative analysis of *ECL_01758*, *ECL_01760 ECL_01761*, *ECL_01762*, *phoP*, *phoQ*, or *arnB* genes differentially expressed in: (A and B) ECL13047 culture with NOSO-502 or CST (32 μg/mL, 30 min) relative to culture without NOSO-502 or CST; (C and D) ECL13047 deletion mutant cultures with NOSO-502 or CST (32 μg/mL, 30 min) relative to ECL13047 culture with NOSO-502 or CST (32 μg/mL, 30 min); (E) culture with NOSO-502 (32 μg/mL, 30 min) of ECC strains belonging to C-XI, and C-XII relative to culture without NOSO-502.

To further explore the role of ECL_01758 efflux system and genes involved in NOSO-502 resistance regulation, we measured by qRT-PCR the expression of genes encoding *ECL_01758*, *ECL_01761*, *ECL_01762*, *phoQ*, *phoP*, and *arnB* in NOSO-502-treated and untreated ECL13047 cultures. Analysis revealed a 6- to 2,600-fold expression increase of these six genes in treated culture compared with untreated one (Fig. 4A). NOSO-502 was therefore able to activate both TCS ECL_01761/62 and PhoPQ.

We then confirmed that the regulation of *ECL_01758* was under the control of *ECL_01761*, and *ECL_01762* by qRT-PCR comparison of the relative mRNA expression of these genes in NOSO-502-treated cultures of ECL13047 deletion mutants and ECL13047. Whereas expression of *ECL_01761* and *ECL_01762* genes remained unchanged in ECL13047 ΔECL_01758 mutant compared with ECL13047, our results demonstrated alterations in the expression of gene *ECL_01758* in ΔECL_01761, and ΔECL_01762 ECL13047 mutants (Fig. 4C). These results clearly showed that in the presence of NOSO-502, *ECL_01760* was also regulated by ECL_01761, and ECL_01762 (Fig. 4C). As expected, in ECL13047, *arn* expression was under the control of PhoQ (Fig. 4C).

### Roles of ECL_01758 and AcrAB-TolC effux pump in ECL13047 resistance to NOSO-502.

The ECL_01758 protein has 49% identity to AcrB protein of ATCC13047 (4). In *Enterobacteriaceae* species, AcrAB-TolC is the most important efflux system involved in both intrinsic and acquired resistance to many antibiotics. Moreover, it has been shown that KexD (ortholog of ECL_01758) functions with AcrA and TolC in K. pneumoniae (27). Guérin et al. described that the *acrB* deletion mutant of ECL13047 was more susceptible to several antibiotics and that *trans*-complementation of this mutant by ECL_01758 restored the wild-type phenotype (4). In order to evaluate the role of AcrB in ECL13047 resistance to NOSO-502 and its possible compensatory effect with ECL_01758, MIC values of compound were first determined against ECL13047Δ*acrB* and ECL13047Δ*acrB*ΔECL_01758 mutants. Unlike other antibiotics, a deletion of *acrB* increased the MIC value of NOSO-502 by an 8-fold factor compared with wild-type strain (MIC = 2,048 μg/mL), while the double mutant Δ*acrB* ΔECL_01758 displayed a decreased of NOSO-502 MIC by a 128-fold factor (MIC = 2 μg/mL), showing that the increased MIC measured against the *acrB* deletion mutant was only due to ECL_01758 but not by another compensating efflux pump (Table 3). Complementation of ECL13047Δ*acrB* by ECL_01758 further increased resistance to NOSO-502 with MIC > 2048 μg/mL (Table 3). These results confirm that AcrB is not directly involved in the resistance of ECL13047 to NOSO-502 but in its absence, the ECL_01758 efflux activity seems to be upregulated.

RT-qPCR was used to quantify the expression of ECL_01758 in NOSO-502-treated versus untreated cultures of ECL13047 and ECL13047Δ*acrB*. Overexpression of ECL_01758 was twice lower in ECL13047ΔacrB culture than in ECL13047 culture (23-fold and 40-fold increase, respectively), excluding an *ECL_01758* gene upregulation in the absence of the *acrB* gene. MIC values of 2 μg/mL and 8 μg/mL against ECL13047Δ*tolC* and ECL13047Δ*acrA* mutants confirmed that ECL_01758 also works with both efflux pump subunits supporting our hypothesis of a functional competition between AcrB and ECL_01758 to function with AcrA and TolC.

### ECL_01760, an ortholog of CrrC, contributes to CST hetero-resistance in ECL13047.

In K. pneumoniae, mutations in the TCS CrrAB upregulates the expression of CrrC, resulting in CST resistance (25).

MIC values of CST were determined against ECL13047 mutants deleted of *ECL_01760*, *ECL_01761*, or *ECL_01762*. A strong reduction of ECL13047 resistance to CST was observed in *ECL_01760* or *ECL_01761* deletion mutants with MIC value of 1 μg/mL while deletion of ECL_01762 did not affect the CST MIC value. Complementation of ECL13047Δ*ECL_01760* and ECL13047Δ*ECL_01761* mutants by *ECL_01760* and *ECL_01761*, respectively, restored resistant phenotypes. Interestingly, using the Etest method, no CST-resistant colonies were observed within the clear zone of inhibition in ECL13047ΔECL_01760 and ECL13047ΔECL_01761 confirming the role of ECL_01760 and ECL_01761 in the CST-hetero-resistant phenotype of ECL13047 (Fig. 2).

As observed with NOSO-502, an upregulation of *ECL_01758*, *ECL_01760*, *ECL_01761*, *ECL_01762*, *phoP*, *phoQ*, and *arnB* gene expressions was measured by qRT-PCR in ECL13047 CST-treated culture compared with the untreated one, confirming that CST was also able to activate both TCS ECL_01761/62 and PhoPQ (Fig. 4B). qRT-PCR analysis revealed a 30-fold decrease of the ECL_01760 gene expression in CST-treated cultures of ECL13047 carrying *ECL_01761* deletion compared with wild-type strain, while only a limited difference (×3) was observed in ECL13047Δ*ECL_01762* mutant culture (Fig. 4D). These results confirm that ECL_01761 acts as positive regulators of ECL_01760 expression but as previously observed with MIC values, ECL_01762 only seems to play a minor role in ECL_01760 regulation. A histidine kinase other than ECL_01762 must therefore regulate ECL_01761 activity but not PhoQ because the Δ*phoQ* mutant maintains levels of ECL_01761 and ECL_01760 expression identical to these observed in the wild-type strain (Fig. 4D).

Interestingly, no significant variation of *phoP*, *phoQ*, or *arnB* gene expression was observed in ECL13047ΔECL_01760 or ECL13047ΔECL_01761 mutants compared with the parental strain (Fig. 4D). It, therefore, means that in both deletion mutants susceptible to CST, the level of *phoPQ* and especially *arn* gene expression remains as high as in the CST-resistant wild strain.

ECL_01760 is predicted to be a transporter protein with four transmembrane domains (Smart for Simple Modular Architecture Research Tool allowing the identification and annotation of genetically mobile domains and the analysis of domain architectures; http://SMART.embl-heidelberg.de).

### Distribution of ECL_01758, ECL_01760, ECL_01761, and neighboring genes among ECC genomes.

**FIG 5:**
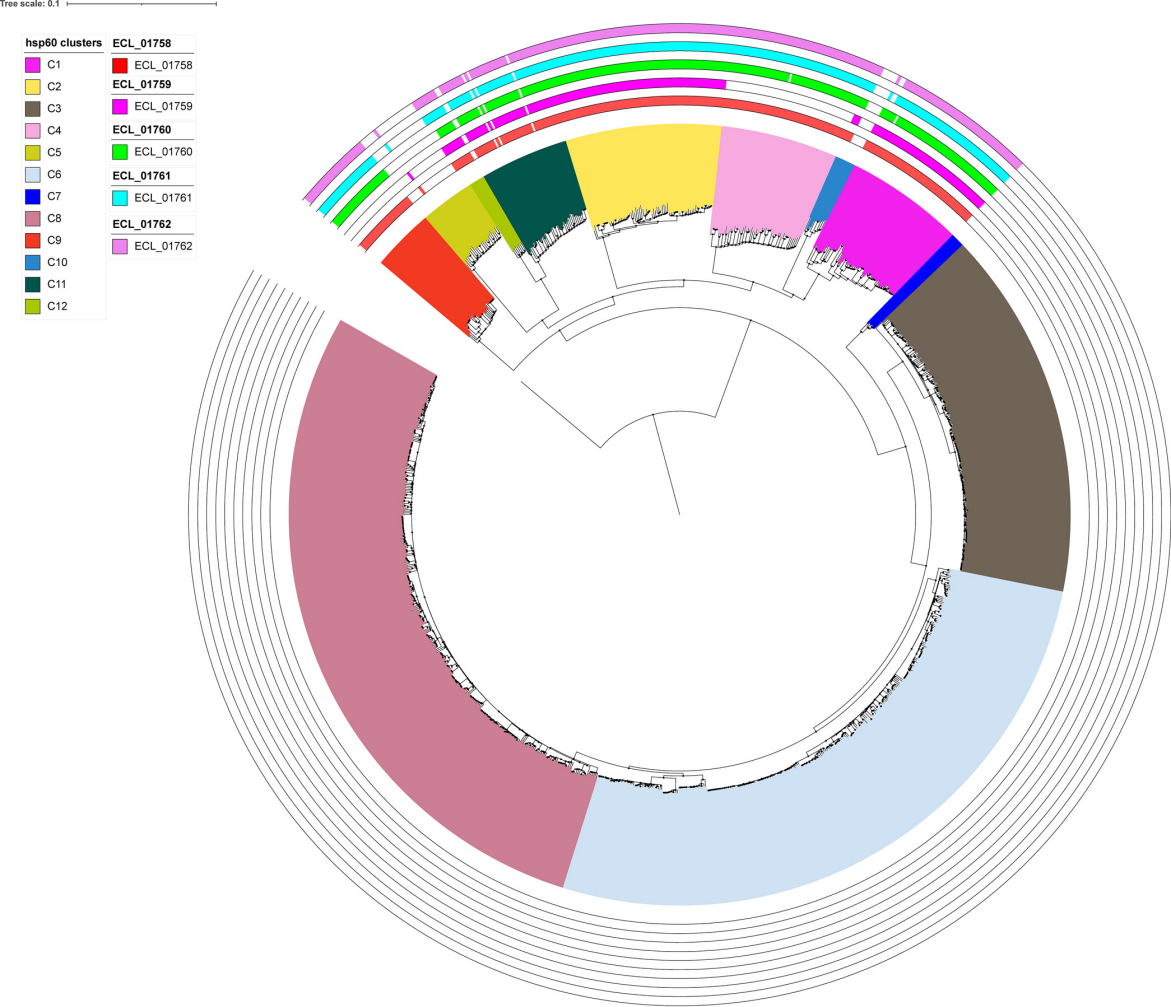
Distribution of ECL_01758, ECL_01759, ECL_01760, ECL_01761, and ECL_01762 genes among 1066 ECC genomes belonging to the 12 Hoffmann clusters. Genes of the ECL_01761/ECL_01762 operon organization and its neighboring genes ECL_01758, ECL_01759, ECL_01760 were looked for using blastn algorithm with “somewhat similar sequences” parameters.

The distribution of *ECL_01758* to *ECL_01762* genes among 1,066 genomes of ECC strains from the different clusters was investigated. Considering an identity and coverage threshold of 50%, all genes were found in genomes of strains belonging to clusters I, II, XI, and XII while C-IV and C-IX have all genes except ECL_01759 (Fig. 5). While the organization of *ECL_01758* to *ECL_01762* genes is highly conserved in C-XI and C-XII strain genomes, we confirmed the absence of the *ECL_01759* gene in C-IV and C-IX isolates and low levels of sequence conservation with ECL13047 in the predicted promoter zone of the operon *ECL_01759-ECL_01758* of C-I and C-II strains, also including the beginning of the *ECL_01759* gene (Fig. 3). The presence of *ECL_01758* to *ECL_01762* genes was evaluated by PCR in the genomes of our 25 ECC clinical isolates from the 12 different clusters. As expected, all genes were only found in genomes of strains belonging to clusters I, II, XI, and XII while *ECL_01759* is the only gene missing in C-IV and C-IX isolates.

The highest NOSO-502 MIC values measured against isolates from clusters C-XI compared with C-XII could be explained by the significative overexpression of the relative mRNA of ECL_01758, ECL_01761, and ECL_01762 genes in treated C-XI strain cultures compared with C-XII strain culture (Fig. 4E).

To better understand the role of ECL_01762 in the NOSO-502 resistance pathway, we *trans*-complemented the C-XII strain RMS39 with *ECL_01762* gene from ECL13047. A 32-fold increase of the NOSO-502 MIC value was observed against RMS39 pBAD202 ΩECL_01762 (MIC = 256 μg/mL) compared with RMS39 (Table 1). This result confirms the influence of ECL_01762 in the NOSO-502 resistance regulation but the mechanism remains unclear.

## DISCUSSION

NOSO-502 is the first preclinical candidate of a novel antibiotic class, the odilorhabdins (ODLs). This cationic peptide inhibits bacterial protein synthesis by targeting the ribosome and it is currently developed to treat infections caused by *Enterobacteriales* such as E. coli, K. pneumoniae, or ECC. In this study, we focused on the characterization of NOSO-502 antibacterial activity against ECC strains that pose a serious threat to human health worldwide due both to the emergence and spread of carbapenem-resistant isolates but also because several of them exhibit hetero-resistance to CST. NOSO-502 demonstrated improved potency, based on MIC values, against a recent and representative population of ECC hospital isolates belonging to all ECC clusters. Intriguingly, only strains from ECC C-XI and C-XII exhibited higher MICs for NOSO-502. To date, isolates of clusters C-XI and C-XII represent only a few cases of ECC isolated from patients. E. cloacae (C-III), *E. hormaechei* subsp. *oharae* (C-VI), and *E. hormaechei* subsp. *steigerwaltii* (C-VIII), including carbapenemase-producing strains, are the most frequent species isolated from intensive care unit (ICU) patients and have previously been identified in a wide variety of nosocomial infections, including cerebral abscess, pneumonia, meningitis, bacteremia, wound, and urinary tract and abdominal cavity/intestinal infections (1, 28). A recent publication reported that in a Spanish hospital between 2005 and 2018, 80% of isolated carbapenemase-producing ECC strains were members of C-III, C-IV, C-VI, or C-VIII, while only 0.5% belong to C-XII. No strains from C-XI were identified (29). Other reports confirm that isolates from cluster XI are rare among carbapenemase-producing ECC isolates (30, 31). Nevertheless, some outbreaks were reported in Togo and the French island of Mayotte (32, 33).

**FIG 6:**
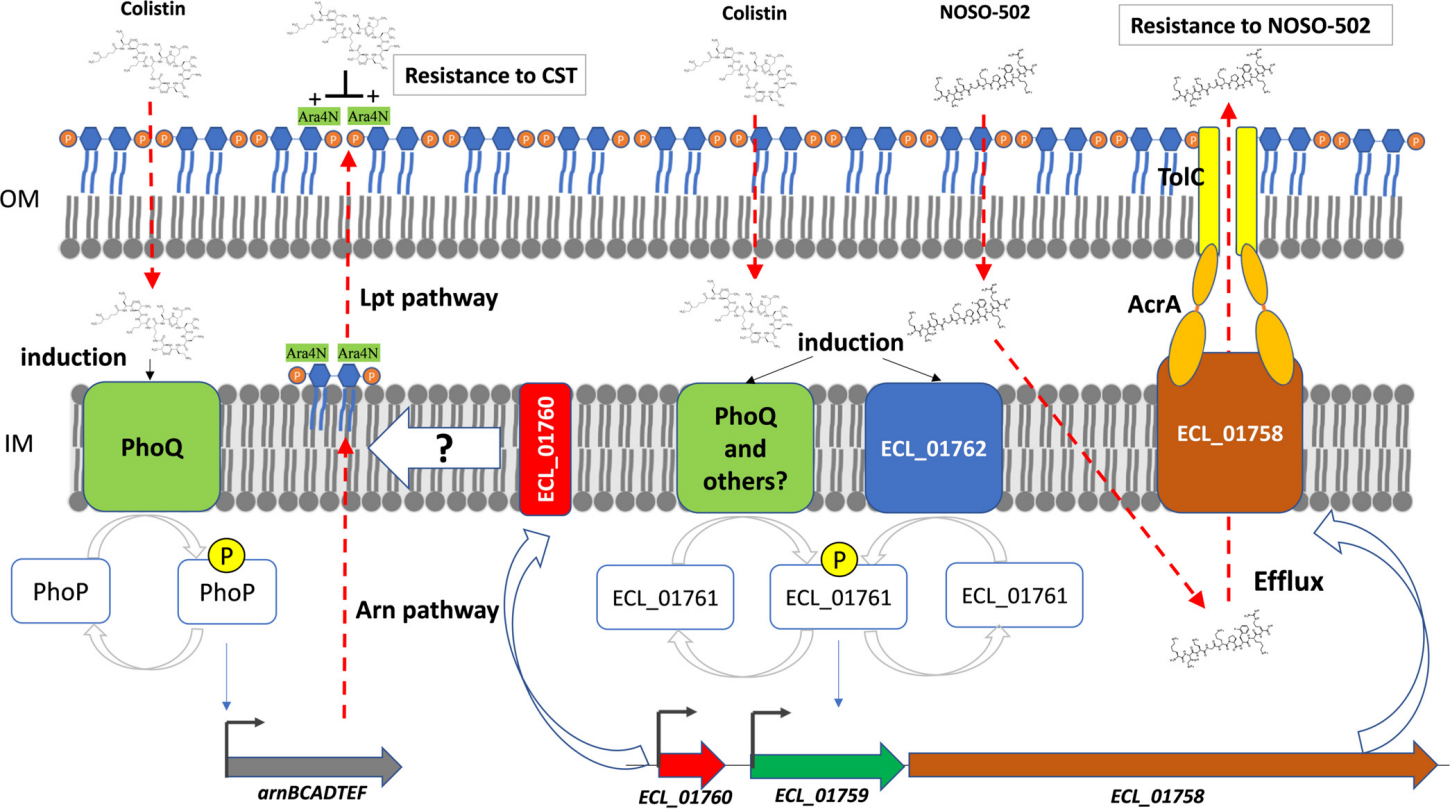
A proposed model of NOSO-502 and colistin resistance in Enterobacter cloacae ATCC 13047.

We clearly demonstrated that the TCS ECL_01761/ECL_01762 plays a pivotal role in the cluster-dependent resistance to NOSO-502 or CST of ECC isolates. In E. cloacae subsp*. cloacae* ATCC 13047 (C-XI), the TCS ECL_01761/ECL_01762 responds directly to the presence of NOSO-502 or CST leading to resistant phenotypes, to NOSO-502 *via* an upregulation of the ECL_01758 efflux pump component but also to CST *via* the overexpression of the transporter protein ECL_01760 (Fig. 6). Nevertheless, the role of the histidine kinase ECL_01762 seems limited in the CST-resistance pathway while that of the response regulator ECL_01761 is preponderant. Many bacteria possess considerable numbers of TCS and the high similarity between some systems raises the possibility of cross talk between a histidine kinase and a noncognate response regulator (34). In this case, another unidentified histidine kinase seems to work with ECL_01761. Increased drug efflux driven by TCS was found across many species of MDR bacteria (35). ECL_01758 complements the hypersusceptibility phenotype of the Δ*acrB* ECL13047 mutant to restore wild-type susceptibility to diverse antibiotics classes such as fluoroquinolones, aminoglycosides, or tetracyclines (4). However, we noted that the inactivation of AcrB increased the resistance of ECL13047 to NOSO-502 while the deletion of *tolC* or *acrA* had the opposite effect, confirming a possible functional competition between ECL_01758 and AcrB to work with AcrA and TolC. In K. pneumoniae, Ogawa and collaborators have already showed that KexD, an ortholog gene of ECL_01758, functions with AcrA and TolC (27). Guérin and collaborators have also identified that the inactivation of ECL_01758 increased the virulence of ECL13047 in a Galleria mellonella model compared with the wild-type strain, while the *acrB* deletion mutant was avirulent (4). This is an important point because it means that cationic peptides like NOSO-502, polymyxin, or AMPs of innate immunity could downregulate the virulence of E. cloacae by induction of ECL_01761/ECL_01762 and by overexpression of ECL_01758. Although only present in clusters I, II, IV, and IX, this tripartite system seems to be solely functional in isolates from C-XI and to a lesser degree from C-XII. It could explain the very low prevalence of C-XI bacterial isolates in human infections. As previously observed in K. pneumoniae, we cannot exclude that mutation in the ECL_01762 gene induces overexpression of the ECL_01758 efflux pump component leading to NOSO-502 resistance in strains belonging to these clusters.

As previously mentioned, the TCS ECL_01761/ECL_01762 led to CST resistance by overexpressing the transporter protein ECL_01760. The presence of CST activates both ECL_01761/ECL_01762 and PhoPQ (Fig. 6). Previous works described CST hetero-resistance in ECL13047 was due to a modification of the LPS, achieved by the addition of L-Ara4N mediated by enzymes of the *arnBCADTEF* operon under the control of the PhoPQ two-component system (16). However, we observed that deletion of ECL_01760 restores full susceptibility to CST, implying that this gene also plays a role in this resistance phenotype. ECL_01760 codes for a small integral membrane protein of 128 amino acids, predicted to be a transporter with four transmembrane domains. It has already been reported that some small proteins connect different TCSs to adapt cells to environmental stress. In Salmonella, the PhoP activated PmrD protein binds to the phosphorylated form of PmrA resulting in the expression of PmrA-regulated LPS modification genes (36). In E. coli, SafA formerly B1500, is a 65 amino-acid membrane protein connecting both TCSs EvgAS and PhoPQ. Its expression is regulated by the EvgAS and this protein directly interacts with PhoQ to activate the PhoPQ system (37, 38). In our study, ECL_01760 acts downstream of the *arnBCADTEF* operon expression and, therefore, of the PhoPQ system. Indeed, similar upregulations of *phoPQ* and *arnB* expression were observed in CST-treated cultures of the CST-susceptible ECL13047 ΔECL_01760 mutant and of the CST-resistant wild-type strain. ArnBCAD enzymes were involved in the biosynthesis of the undecaprenyl phosphate-α-l-Ara4N while ArnEF ensure its transport across the inner membrane. These steps precede periplasmic modification of lipid A with l-Ara4N by the undecaprenyl phosphate-alpha-4-amino-4-deoxy-l-arabinose arabinosyl transferase ArnT. ECL_01760 could, therefore, act on the inner membrane transport of the undecaprenyl phosphate-α-l-Ara4N cytoplasmic precursor (Fig. 6). These results seem different from those observed in K. pneumoniae by Cheng et al. who demonstrated that CrrC, an ortholog gene of ECL_01760, regulated the *pmrHFIJKLM* operon, also called *arnBCADTEF*, and *pmrC* through the PmrAB TCS (25). Interestingly, in K. pneumoniae, the small protein DkcA, displays similarity to membrane transporter and is required for CST resistance in a lipid A-dependent and-independent manner (39). DkcA is required for lipid A modification with Ara4N by a yet unidentified mechanism, but authors hypothesized that it would play a role in the *arn* pathway or by maintaining an inner membrane potential necessary for the CST antibacterial activity.

### Conclusion.

NOSO-502 demonstrated potent *in vitro* activity against clinically relevant ECC associated with different infections in humans. Only isolates belonging to clusters XI and XII, rarely found in clinical cases, exhibited decreased susceptibility to NOSO-502. This study revealed that in the C-XI strain ECL13047, the TCS ECL_01761-ECL_01762 is induced by NOSO-502 or CST and regulates NOSO-502 hetero-resistance by an overexpression of the ECL_01758 efflux pump component and CST resistance by the overexpression of the ECL_01760 putative transporter (Fig. 6).

## MATERIALS AND METHODS

### Bacterial strains.

A total of 94 reference, clinical, and mutant strains were included in this study. Fifty clinical isolates (C1.301 to C1.350) were isolated from patients hospitalized in United Kingdom during the last 3 years. An additional 25 clinical isolates from several French hospitals were obtained from different sources. These ECC clinical isolates were allocated to their genetic clusters by *hsp60* partial sequence analysis carried out as previously described (40). The reference strain used in the study was E. cloacae subsp. *cloacae* ATCC 13047 (belonging to the cluster XI). This strain was isolated from human cerebrospinal fluid and corresponds to the type strain of E. cloacae subsp. *cloacae* (26). It was also the first reference strain fully sequenced and annotated (GenBank accession numbers CP002886, FP929040, and AGSY00000000) (26). Mutant strains, derived from E. cloacae subsp. *cloacae* ATCC 13047, used in this study are listed in Table 4.

**TABLE 4:** Strains and plasmid used in this study

Strains or plasmid	Characteristic(s)	Reference
Strains		
E. cloacae ATCC13047 (ECL13047)	Reference strain	26
ECL13047ΔECL_02504 (*phoP*)	Deleted ECL_02504	17
ECL13047ΔECL_02505 (*phoQ*)	Deleted ECL_02505	17
ECL13047ΔECL_02504-5 (*phoPQ*)	Deleted ECL_02504-5	17
ECL13047ΔECL_04562 (*pmrA*)	Deleted ECL_04562	17
ECL13047ΔECL_04563 (*pmrB*)	Deleted ECL_04563	17
ECL13047ΔECL_04562-3 (*pmrAB*)	Deleted ECL_04562-3	17
ECL13047ΔECL_04857-63 (*arnBCADTEF*)	Deleted ECL_04857-63	17
ECL13047ΔECL_04868 (*arnE*)	Deleted ECL_04868	17
ECL13047ΔECL_04867 (*arnF*)	Deleted ECL_04867	17
ECL13047ΔECL_01233 (*acrB*)	Deleted ECL_01233	4
ECL13047ΔECL_01234 (*acrA*)	Deleted ECL_01234	This study
ECL13047ΔECL_01758 (*kexD*)	Deleted ECL_01758	4
ECL13047ΔECL_01759	Deleted ECL_01759	This study
ECL13047ΔECL_01760 (*crrC*)	Deleted ECL_01760	This study
ECL13047ΔECL_01761 (*crrA*)	Deleted ECL_01761	This study
ECL13047ΔECL_01762 (*crrB*)	Deleted ECL_01762	This study
ECL13047ΔECL_01762 ΔECL_02505	Deleted ECL_01762 & ECL_02505	This study
ECL13047ΔECL_04363 (*tolC*)	Deleted ECL_04363	This study
ECL13047ΔECL_01233 ΔECL_01758	Deleted ECL_01233 & ECL_01758	This study
ECL13047_pBAD202	13047 *trans*-complemented strain carrying pBAD202	This study
ECL13047ΔECL_01758 pBAD202ΩECL_01758	13047_ΔECL_01758 *trans*-complemented strain carrying pBAD202/D-TOPOΩECL_01758	4
ECL13047ΔECL_01759 pBAD202ΩECL_01759	13047_ΔECL_01759 *trans*-complemented strain carrying pBAD202/D-TOPOΩECL_01759	This study
ECL13047ΔECL_01760 pBAD202ΩECL_01760 (*crrC*)	13047_ΔECL_01760 *trans*-complemented strain carrying pBAD202/D-TOPOΩECL_01760	This study
ECL13047ΔECL_01761 pBAD202ΩECL_01761 (*crrA*)	13047_ΔECL_01761 *trans*-complemented strain carrying pBAD202/D-TOPOΩECL_01761	This study
ECL13047ΔECL_01762 pBAD202ΩECL_01762 (*crrB*)	13047_ΔECL_01762 *trans*-complemented strain carrying pBAD202/D-TOPOΩECL_01762	This study
ECL13047ΔECL_01233 pBAD202	13047_ΔECL_01233 *trans*-complemented strain carrying pBAD202	
ECL13047ΔECL_01233 pBAD202ΩECL_01758	13047_ΔECL_01233 *trans*-complemented strain carrying pBAD202/D-TOPOΩECL_01758	
RMS39	Clinical isolate belonging to cluster XII	This study
RMS39 pBAD202	ECL_146 *trans*-complemented strain carrying pBAD202	This study
RMS39 pBAD202 ΩECL_01762 (*crrB*)	ECL_146 *trans*-complemented strain carrying pBAD202/D-TOPOΩECL_01762	This study
Plasmid		
pBAD202	General expression vector with arabinose-inducible promoter, Kanamycin	Life Technologies
pKD4	Plasmid containing an FRT-flanked kanamycin cassette, Kanr	45
pCP20	Ampicillin and Cm^r^ plasmid that shows temp-sensitive replication and thermal induction of FLP synthesis	46
pKOBEG	Recombination vector, phage λ recγβα operon under the control of the pBAD promoter, Cm^r^	51

### Antimicrobial agents and media.

NOSO-502 was synthesized at Nosopharm, Nîmes, France (Biosynth, ref: AC20542), ciprofloxacin (Biosynth, ref: AC58172), meropenem (Biosynth, ref: AM32026), ceftazidime-avibactam (CAZ: Biosynth, ref: AC19871; AVI: Biosynth, ref: AA158833), tigecycline (Biosynth, ref: AT10818), and amikacin (CAZ: Biosynth, ref: AA17356) were obtained from manufacturers as standard powders. The BBL Mueller-Hinton II Broth (Becton, Dickinson, ref: 212322), alone or with 1.4% wt/wt agarose (Grosseron ref A8963) and the Mueller-Hinton Agar (Oxoid, ref CM0337) were used in all experiments. When necessary, Mueller-Hinton was supplemented with kanamycin at 40 μg/mL (Sigma-Aldrich, ref: K1637).

### Minimum inhibitory concentration (MIC).

MIC values were determined using Clinical and Laboratory Standards Institute (CLSI) broth microdilution (BMD) methodology, colony direct suspension, as described in CLSI document M07-A10 (41).

### DNA extraction.

Genomic DNA extraction from bacterial cultures was conducted using the Qiagen QIAamp DNA minikit ref. 51304, according to manufacturer protocols. All DNA preparations were kept at −20°C until use.

### ECC cluster membership characterization.

ECC clinical isolates were allocated to their genetic clusters by *hsp60* sequence analysis carried out as previously described (40). The *hsp60* gene was amplified by PCR using the Platinum *Taq* polymerase High Fidelity (Invitrogen) and with the primers hsp60-F and hsp60-R (Table S3). PCR was conducted on a Mastercycler thermocycler (Eppendorf) under the following conditions: initialization 7 min at 94°C, followed by 35 cycles: 30 s at 94°C, 30 s at 65°C, and 30 s at 68°C, and a final extend step of 7 min at 68°C. Both strands of the purified PCR product were sequenced using a commercial sequencing service (Eurofins, Ebersberg, Germany). Nucleotide sequences of *hsp60* used in this study for ECC analysis were retrieved from GenBank. ECC strains were allocated to their genetic clusters according to the *hsp60* partial sequence protocol using BLAST (Basic Local Alignment Search Tool: https://blast.ncbi.nlm.nih.gov/).

### Detection of orthologs genes from ECL_01758 to ECL_01762 genes.

The presence of ECL_01758 and ECL_01762 genes was determined through PCR using primers listed in Table S3 and under the following conditions: initialization 2 min at 94°C, followed by 35 cycles: 15 s at 94°C, 20 s at 62°C, and 1 min 10s at 68°C, final extend step of 5 min at 68°C.

### Population analysis profile.

Population analysis profiling was performed by plating serial saline dilutions of an overnight fresh culture onto Mueller-Hinton agarose containing 1 to 256 μg/mL NOSO-502 or CST (in 2-fold increments). Plates were incubated overnight at 35°C and frequency of the subpopulation was determined at 24 h and 48 h of incubation by dividing by the total number of cells (42).

### Time-dependent killing.

Experiment was performed according to CLSI guidelines for determining bactericidal activity of NOSO-502 (43). Cultures were conducted into 6-wells microplate (Sarstedt ref. 83.3920.500).

### Etest assay.

The inoculum was realized by picking approximately 10 colonies from an overnight streaking on Mueller-Hinton agar, preparing a suspension in 5 mL of saline until OD_600nm_ reached 0.15 to 0.2, and vortexing the suspension for 15 s. Mueller-Hinton agarose plates were poured in order to get 4 mm of thickness and inoculated using a swab. Plates were dried 20 min before spotting 10 μL of each solution at 100× or applying the Etest strip. Plates were incubated at 35°C in a loosely folded plastic bag to maintain moisture.

### Determination of mRNA expression levels by RT-qPCR.

Bacterial culture in CAMHB at OD_600nm_ comprised between 0.5 and 1. Total mRNA extraction was achieved with the RNeasy Protect Bacteria 50 preps kit (Qiagen ref. 74524) according to the manufacturer’s instructions and was performed on three independent biological replicates.

RNA Integrity Number (RIN) were determined, and reverse transcription was performed using SuperScript II Reverse Transcriptase (Invitrogen ref. 18064-022) and random hexamer from Applied Biosystems ref. N8080127.

RT-qPCR to follow *phoP*, *phoQ*, ECL_01761, ECL_01762, ECL_01760, *acrB*, and ECL_01758 gene expressions was carried out using a LightCycler 480 (Roche) with Sensi-Fast SYBR No-ROX commercialized by Bioline (BIO-98050) and qPCR primers listed in Table S3. The experiment was performed in triplicates on each cDNA sample.

As control, a blank sample (distilled water) and a no reverse transcriptase control were included to exclude DNA contamination. The *rpoB* gene was used as the reference housekeeping gene.

The data for each sample are expressed relative to the level of *rpoB*, using REST software 2009 and the Pfaffl equation (44).

This method quantified the expression of target genes relative to that of a reference gene, for comparisons of parental strains E. cloacae ECL13047 with mutants in the presence of NOSO-502 at subinhibitory concentrations for 30 min or in absence as control.

### Construction of knockout deletion mutants.

Disruption of the selected genes was performed using the method described by Datsenko and Wanner with some modifications, using the plasmid pKOBEG as previously described (45, 46).

### Construction of a multicopy plasmid library containing putative genes of interest or regulator open reading frames.

The regulator and the genes of interest, including their own promoters, were amplified by PCR using primers listed in Table S3. Each amplicon was then TA cloned into the pBAD202 Directional TOPO overexpression plasmid (low-copy-number plasmid, ~20 copies/cell; Invitrogen, Villebon sur Yvette, France). E. coli TOP-10 cells (Invitrogen) carrying pBAD202 recombinants containing correctly oriented inserts were selected on LB plates with 40 mg/L of kanamycin. After purification, each plasmid carrying the regulator or genes of interest was used to transform the ECL13047 strain and clinical isolates (Table 4).

### *In silico* analyses.

Genomes of Enterobacter cloacae strains have been downloaded from two databases, NCBI RefSeq (https://www.ncbi.nlm.nih.gov/refseq) and Patric database (https://www.patricbrc.org/). The quality of the genome assemblies was evaluated using Quast software (47). Then, species determination was performed using the PGAP algorithm (48). Only genomes with less than 300 contigs and which belonged to the Enterobacter cloacae species with a high degree of confidence were conserved. A total of 1,066 genomes were included for further investigations. Information about the selected strains is available in Table S4. Hsp60 cluster attribution were *in silico* performed using sequences extracted from GenBank. The genes ECL_01758, ECL_01759, ECL_01760 as well as ECL_01761/ECL_01762 operon were looked for using blastn algorithm with the following parameters: word size 11, gap open 5, gap extend 2, reward 2 and penalty −3.

Genomic distance between the 1,066 genomes was assessed using the mash triangle algorithm, then analyzed using neighbor for the PHYLIP package generating an unroot Neighbor Joining tree (49). The phylogenetic tree and the blast results was illustrated using iTOL (50).
