# Supplementary material for: Exploring Cluster-Dependent Antibacterial Activities and Resistance Pathways of NOSO-502 and Colistin against Enterobacter cloacae Complex Species
Source: Antimicrob Agents Chemother. 2022 Oct 6;66(11):e00776-22. doi: 10.1128/aac.00776-22 (PMC9664853; doi:10.1128/aac.00776-22)
Supplement: Supplemental file 1 — Fig. S1 and S2 and Tables S1 to S3. Download aac.00776-22-s0001.pdf, PDF file, 0.7 MB [file aac.00776-22-s0001.pdf]

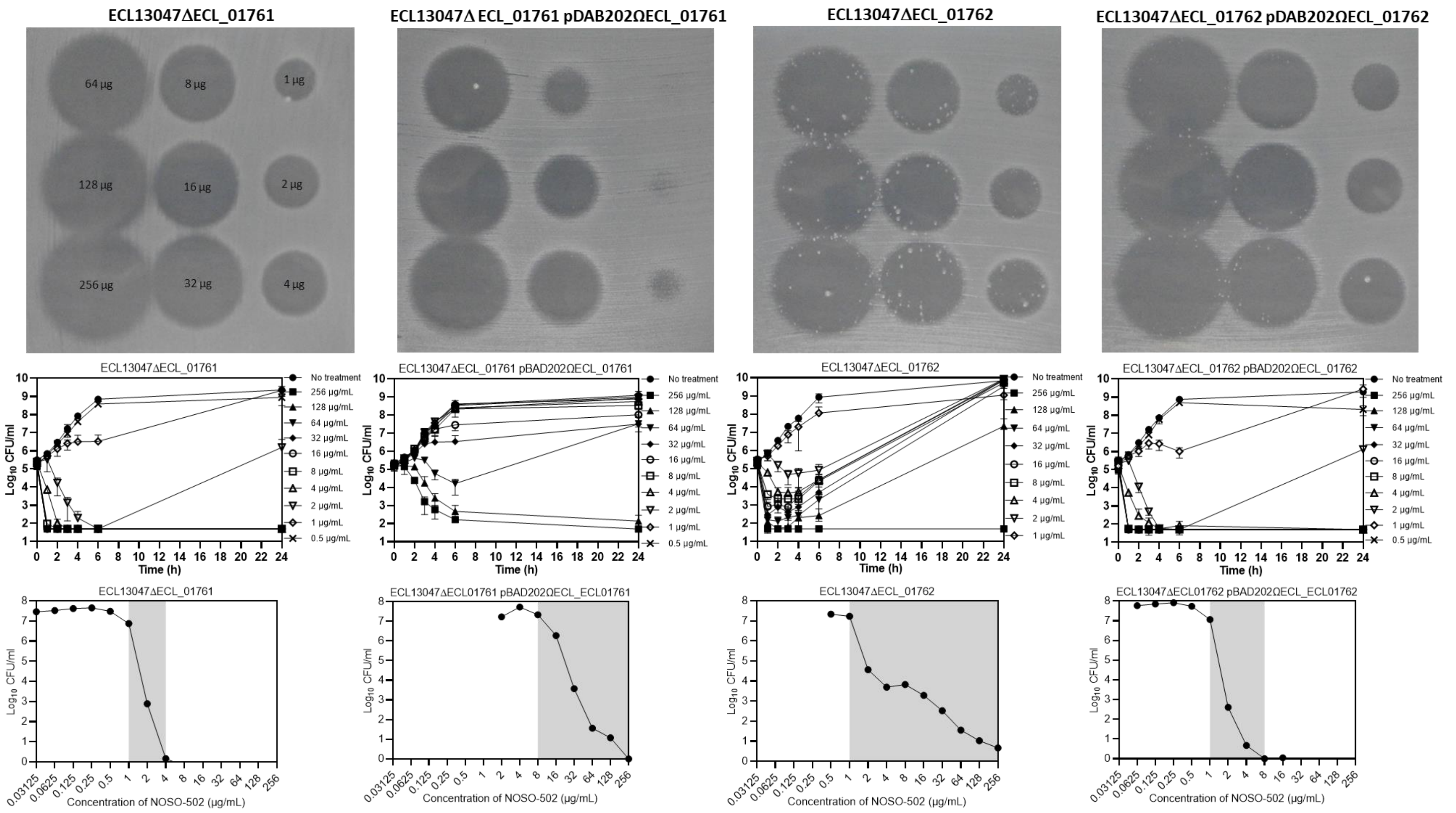

Figure S1: Determination of NOSO-502 antibacterial activity and hetero-resistance in ECL13047ΔECL_01761, ECL13047ΔECL_01762, ECL13047ΔECL_01761 pBAD202ΩECL_01761, and ECL13047ΔECL_01762 pBAD202ΩECL_01762 strains by (A) Agar diffusion assay with NOSO-502 at 1 to 256 µg; (B) Time-kill study with NOSO-502 at 0.5 to 256 µg/mL; (C) Population analysis profile (PAP) method.

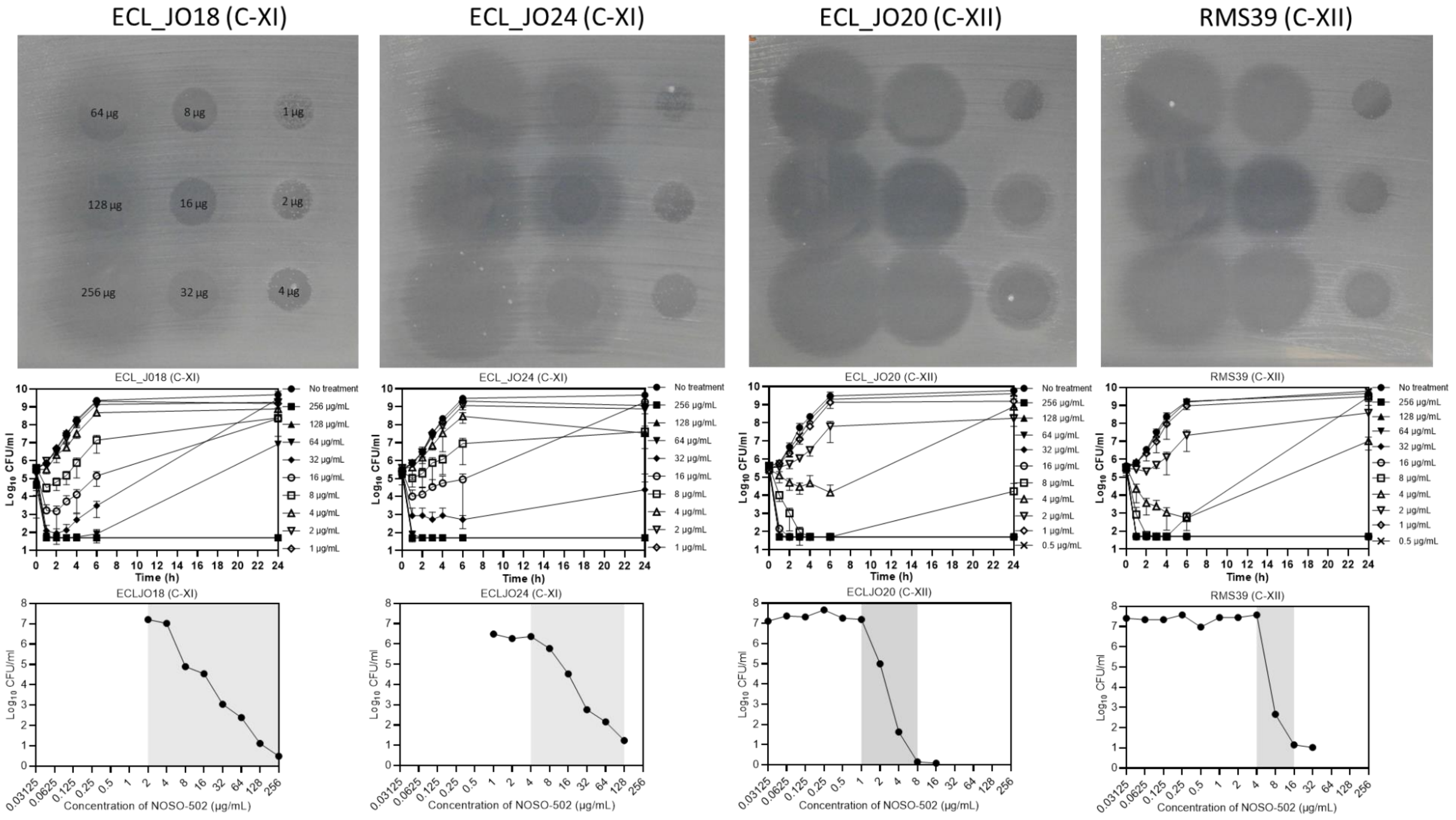

Figure S2: Determination of NOSO-502 antibacterial activity and hetero-resistance in ECL_JO18 (C-XI), ECL_JO24 (C-XI), ECL_JO20 (C-XII), and RMS39 (C-XII) strains by (A) Agar diffusion assay with NOSO-502 at 1 to 256 µg; (B) Time-kill study with NOSO-502 at 0.5 to 256 µg/mL; (C) Population analysis profile (PAP) method.

Enterobacter asburiae	I
Enterobacter kobei	II
Enterobacter cloacae complex Hoffmann cluster III	III
Enterobacter cloacae complex Hoffmann cluster IV	IV
Enterobacter ludwigii	V
Enterobacter xiangfangensis	VI
Enterobacter hormaechei ssp. hormaechei	VII
Enterobacter hormaechei ssp. steigerwaltii	VIII
Enterobacter bugandensis	IX
Enterobacter nimipressuralis	X
Enterobacter cloacae ssp. cloacae	XI
Enterobacter cloacae ssp. dissolvens	XII

Table S1: Hoffmann's cluster classification according to the *hsp60* gene sequencing.

ECC species	Strain	Site of infection	Year of isolation	MIC (µg/mL)				Antibiogram								
				NOS	CZA	CST	TGC	AMC	CIP	GEN	MEM	TZP	AMK	ATM	CEF	TOB
E. xiangfangensis	C1.333	UNK	<3	1	>16	0.05	2	R	R	R	R	R	R	nd	nd	R
E. xiangfangensis	C1.334	UNK	<3	1	16	0.05	1	R	R	R	R	R	R	R	nd	R
E. xiangfangensis	C1.336	BCL	<3	1	>16	0.05	1	R	S	S	S	R	S	R	S	S
E. xiangfangensis	C1.337	BCL	<3	1	>16	0.05	1	R	S	S	S	R	S	R	S	S
E. xiangfangensis	C1.301	BCL	<3	1	4	0.25	1	R	S	S	S	S	S	S	S	S
E. cloacae	C1.302	BCL	<3	1	4	0.25	1	R	S	S	S	S	S	S	S	S
E. cloacae	C1.303	BCL	<3	1	1	0.25	0.5	R	S	S	S	S	S	S	S	S
E. cloacae	C1.308	TIS	<3	1	2	0.25	0.5	R	S	S	S	S	S	S	S	S
E. cloacae	C1.314	FLD	<3	1	2	0.25	0.5	R	S	S	S	S	S	S	nd	S
E. cloacae	C1.315	TIS	<3	1	2	0.25	1	R	S	S	nd	S	S	S	S	S
E. cloacae	C1.318	TIS	<3	1	0.12	0.25	1	R	S	S	S	S	S	S	S	S
E. xiangfangensis	C1.319	TIS	<3	1	4	0.25	1	R	S	S	S	S	S	S	S	S
E. cloacae	C1.322	BCL	<3	1	1	0.25	1	R	S	S	S	S	S	S	S	S
E. cloacae	C1.325	BCL	<3	1	1	0.25	0.5	R	I	S	S	S	S	S	S	S
E. cloacae	C1.339	BCL	<3	1	>16	0.25	4	R	S	S	S	S	S	R	I	S
E. ludwigii	C1.340	BCL	<3	1	>16	0.25	0.5	R	S	S	nd	nd	S	R	S	S
E. ludwigii	C1.341	PFLD	<3	1	>16	0.25	0.5	R	S	S	nd	S	S	R	S	S
E. cloacae	C1.344	BCL	<3	1	8	0.25	2	R	S	S	S	I	S	S	S	S
E. xiangfangensis	C1.348	TIS	<3	1	4	0.25	0.5	R	S	S	S	R	S	R	I	S
E. xiangfangensis	C1.326	BCL	<3	2	>16	0.25	2	R	S	R	S	S	S	S	I	R
E. cloacae	C1.338	BCL	<3	2	>16	0.25	0.5	R	S	S	S	I	S	I	S	S
E. cloacae	C1.347	FLD	<3	0.5	>16	0.5	0.5	R	S	S	S	R	S	R	I	S
E. cloacae	C1.305	BCL	<3	1	0.5	0.5	0.5	R	S	S	S	S	S	S	S	S
E. cloacae	C1.306	BCL	<3	1	1	0.5	0.5	R	S	S	S	S	S	S	S	S
E. cloacae	C1.307	BCL	<3	1	0.12	0.5	0.5	R	S	S	S	S	S	S	S	S
E. cloacae	C1.309	BCL	<3	1	1	0.5	2	R	S	S	S	S	S	S	S	S
E. cloacae	C1.311	TIS	<3	1	1	0.5	0.5	R	S	S	S	S	S	S	S	S
E. cloacae	C1.312	TIS	<3	1	1	0.5	0.5	R	S	S	S	S	S	S	S	S
E. cloacae	C1.313	TIS	<3	1	0.25	0.5	1	R	S	nd	nd	S	S	S	S	S
E. xiangfangensis	C1.321	TIS	<3	1	2	0.5	0.5	R	S	S	nd	S	S	S	S	S
E. cloacae	C1.323	TIS	<3	1	2	0.5	1	R	S	S	nd	S	S	S	S	S
E. cloacae	C1.327	BCL	<3	1	16	0.5	1	R	S	S	S	S	S	R	S	S

E. cloacae	C1.330	BCL	<3	1	>16	0.5	1	nd	nd	nd	S	nd	nd	S	R	nd
E. cloacae	C1.331	WD	<3	1	16	0.5	0.5	R	S	S	R	R	nd	S	nd	nd
E. xiangfangensis	C1.332	UNK	<3	1	>16	0.5	0.5	nd	nd	I	R	nd	R	nd	nd	R
E. cloacae	C1.342	TIS	<3	1	>16	0.5	0.5	R	S	S	S	R	S	R	S	S
E. cloacae	C1.343	BCL	<3	1	>16	0.5	0.5	R	S	S	S	R	S	R	nd	S
E. cloacae	C1.345	BCL	<3	1	8	0.5	0.5	R	S	S	S	S	S	S	S	S
E. cloacae	C1.349	TIP	<3	1	>16	0.5	2	R	S	S	S	R	nd	nd	nd	Nd
E. cloacae	C1.350	TIS	<3	1	>16	0.5	1	R	S	S	S	S	nd	nd	nd	nd
E. cloacae	C1.346	BCL	<3	2	4	0.5	0.5	R	S	S	S	S	S	S	S	S
E. cloacae	C1.324	BCL	<3	1	2	<0.12	0.5	R	S	S	S	S	S	S	S	S
E. cloacae	C1.328	BCL	<3	1	16	<0.12	1	R	S	R	S	S	S	R	R	R
E. cloacae	C1.304	BCL	<3	1	1	4	0.5	R	S	S	S	S	nd	nd	nd	nd
E. bugandensis	C1.310	TIS	<3	1	4	8	1	R	S	S	nd	S	S	S	S	S
E. cloacae	C1.329	BCL	<3	1	>16	16	2	R	S	S	S	R	S	R	S	S
E. bugandensis	C1.317	BCL	<3	2	4	16	1	R	S	S	S	S	nd	nd	nd	nd
E. bugandensis	C1.316	BCL	<3	1	1	>64	1	R	S	S	S	S	S	S	S	S
E. cloacae	C1.335	BCL	<3	16	>16	>64	2	R	S	S	S	R	S	R	S	S
E. cloacae	C1.320	TIS	<3	32	2	>64	1	R	S	S	nd	S	S	S	S	S

Table S2: MIC of NOSO-502 and antimicrobial agents against a panel of recent ECC isolates from United Kingdom. Abbreviations: UNK: unknown, BCL: blood culture, TIS: tissue; FLD: fluid, PFLD: pleural fluid, WD: wound, TIP: Line tip, NOS: NOSO-502, CZA: ceftazidime/avibactam, CST: colistin, TGC: tigecycline, AMC: amoxicillin + clavulanic acid, CIP: ciprofloxacin, GEN: gentamicin, MEM: meropenem, TZP: piperacillin + tazobactam, AMK: amikacin, ATM: aztreonam, CEF: cefepime, TOB: tobramycin, R: resistant, S: susceptible, I: intermediate, nd: not determined .

Primer	Nucleotide sequence (5’ to 3’)	*Purpose*
ECL_01234-pKD4-F	CGGACACTCGAGGTTTACATATGAACAAAAACAGAGGGTTAACGCCTCTGGCGGGTGTAGGCTGGAGCTGCTTC	ECL_01234 deletion
ECL_01234-pkD4-R	CTCCTGTTTAAGTTAAGACTTGGTTTGTTCTGACTGGCCAGCGGCCGAAGCTTGCATATGAATATCCTCCTTAG	
ECL_01759-pKD4-F	CGCTCAAGGCACTTGCCGTGCGGCACCGCAAGAAGCTGTTTATTACCTTCTTTCTGGGTGTAGGCTGGAGCTGCTTC	ECL_01759 deletion
ECL_01759-pkD4-R	GCGGCATGAGGATTACTTCCAGTCAACTAACAGTGAAACGCATTACGGGTGCAGCATATGAATATCCTCCTTAG	
ECL_01760-pKD4-F	GCGATTCAACATAAAGGAGGCGGATTGACGGAACTCCTTGTCATGGCCCTGGTGTAGGCTGGAGCTGCTTC	ECL_01760 deletion
ECL_01760-pkD4-R	GTGGAGGTTATATGGAGACAGTAAATTAAATCATCCTTAACATGAGCATATGAATATCCTCCTTAG	
ECL_01761-pKD4-F	GCTTTCAGTTCCTGTGGAGATAAAATGTACAAAAATAATTTAATCCTAGTCGGTGTAGGCTGGAGCTGCTTC	ECL_01761 deletion
ECL_01761-pkD4-R	GCGGCTCAGAACAGATTCTCTGCTCATTTCTTTTCTCCCAGCCGGTAGCATATGAATATCCTCCTTAG	
ECL_01762-pKD4-F	GGGCTACCGGCTGGGAGAAAAGAAATGAGCAGAGAATCTGTTCTGAGCCGCCGTGTAGGCTGGAGCTGCTTC	ECL_01762 deletion
ECL_01762-pkD4-R	GCAAACAGGCTCATTTGAGCCTGTACGCAGTTACGACGCGATCGGCCAGGCATATGAATATCCTCCTTAG	
ECL_01760-pKD4-F	GCGATTCAACATAAAGGAGGCGGATTGACGGAACTCCTTGTCATGGCCCTGGTGTAGGCTGGAGCTGCTTC	ECL_01760 deletion
ECL_01760-pkD4-R	GTGGAGGTTATATGGAGACAGTAAATTAAATCATCCTTAACATGAGCATATGAATATCCTCCTTAG	
ECL_04563-pKD4-F	CTGCTTCACAACAAGGAATGCAAATGAAGAAATTGCTCCCCATCCTTATCGGCGTGTAGGCTGGAGCTGCTTC	ECL_04563 deletion
ECL_04563-pkD4-R	GCATCAGGCCGCAGTGGGCCTGATGACACTCTTAGTGACGGAACGGATTGCCATATGAATATCCTCCTTAG	
ECL_01234v_F	TCGTGCCATAAGTGGGTGAA	Verification of construction of Δ01234
ECL_01234v_R	GGATATTGCGCAACAGGCAG	
ECL_01759v_F	ATTGTCCTCCGGTCGTTTCC	Verification of construction of Δ01759
ECL_01759v_R	GTGCAACGACCGGATATTGC	
ECL_01760v_F	TGCATCTTTGGATGATCCTG	Verification of construction of Δ01760
ECL_01760v_R	CGACCGGAGGACAATGAGTA	
ECL_01761v_F	CGTGCGTGGGTCTTAGACTT	Verification of construction of Δ01761
ECL_01761v_R	GCGGCTCAGAACAGATTCTC	
ECL_01762v_F	TCTTCATGGTGTGCCAGAAA	Verification of construction of Δ01762
ECL_01762v_R	GTGAAATGTGGCAAATGACG	
ECL_04563v_F	GCACATTACGCCAACCTTTT	Verification of construction of Δ04563
ECL_04563v_R	GGCTTACGTTCGGATGTGAT	
ECL_01759pBAD202_F	CACCCCTGACGGAATTCCCCTGCTCTC	Construction pBAD202Ω01759
ECL_01759pBAD202_R	TTACTTCCAGTCAACTAACAGTG	
ECL_01760pBAD202_F	CACCCTTCATCTTCGGCGACTAGG	Construction pBAD202Ω01760
ECL_01760pBAD202_R	CGACCGGAGGACAATGAGTA	
ECL_01761pBAD202_F	CACCTGTCGTGCGTGGGTCTTAGACTT	Construction pBAD202Ω01761
ECL_01761pBAD202_R	GCTCAGAACAGATTCTCTGC	
ECL_01762pBAD202_F	CACCTGGAGCTTGCAGGTCTTCATG	Construction pBAD202Ω01762
ECL_01762pBAD202_R	GGCTCATTTGAGCCTGTACGC	
ECL_04563pBAD202_F	CACCCATGGCACATTACGCCAACCT	Construction pBAD202Ω04563
ECL_04563pBAD202_R	CGCAGTGGGCCTGATGACAC	
All clusters		
ECL_rpoB_L	AAGGCGAATCCAGCTTGTTCAGC	qRT-PCR. Quantification of rpoB (Housekeeping gene)
ECL_rpoB_R	TGACGTTGCATGTTCGCACCCATCA	
Cluster XI		
ECL_01758_L	TGGTATAGTCATTAACGTAACTGGAGC	qRT-PCR
ECL_01758_R	TTGATCGTCCTGTTTTCGCC	
ECL_01759_L	CAATCAGTAAATATCCCCACGCTTC	qRT-PCR
ECL_01759_R	TTATGCTGCTGACCATTGAATTCTG	

ECL_01760_L	AATCCGCCTCCTTTATGTTGAATC	qRT-PCR
ECL_01760_R	GTTTGTTTCTTATTGTATCGTGGGG	
ECL_01761_L	AAATCGCCGATATACTGATGAGCTA	qRT-PCR
ECL_01761_R	CAATCCCATCAGCTTATCAACATCC	
ECL_01762_L	GACAGTCAACGTAAATCCTGGAATG	qRT-PCR
ECL_01762_R	AACTGACAACCCTGAGATCTTCTATC	
ECL_phoP_L	GGAAGATGCAAAAGAAGCCGATTAT	qRT-PCR
ECL_phoP_R	TCAATATGAAACGGTTTGGTGACG	
ECL_phoQ_L	CAAATTGGCTACTATCTTCATCGCG	qRT-PCR
ECL_phoQ_R	GAACTCCAGACAATATTTACAGGCG	
ECL_acrB_L	GGGTGTTTATCGAGTAGCCAAATATTG	qRT-PCR
ECL_acrB_R	GTTAAAACCCTGGTAGAAGCGATTATC	
ECL_arnB_L	TTAATGGCGTGGAAGGAGAAAATTG	qRT-PCR
ECL_arnB_R	GATCGATGTGGACAAAGATACCCT	
**Cluster I**		
ECL_01758_L_CI	TGTTCAGACATTTGTGGAAGCAATG	PCR
ECL_01758_R_CI	TGGCATCATCAACGATAATCCCTAT	
ECL_01759_L_CI	CCTGATGGTACTTTGTATGTGGTTC	PCR
ECL_01759_R_CI	GAAGGTGCATTTCAAAGAAATCCAC	
ECL_01760_L_CI	TGTCGGTTGTCTCTCTGATAATACA	PCR
ECL_01760_R_CI	AACACTCGTAGTAAAAGACCAGATC	
ECL_01761_L_CI	GGGTTGAAGGGTTTGATTACGTAAT	PCR
ECL_01761_R_CI	CCTCCATAAACCGGATCTCATATTG	
ECL_01762_L_CI	CGAGGTTGAATAGTGTAAGGCATTA	PCR
ECL_01762_R_CI	GAACGGATCTAAAAGGGACAATACA	
**Cluster IV**		
ECL_01758_L_CIV	TGAAACTGTCTATCATGAAGGTGGT	PCR
ECL_01758_R_CIV	TGGCATCATCAACGATAATCCCTAT	
ECL_01760_L_CIV	GGGATTACTTTATACCTGGCTGATG	PCR
ECL_01760_R_CIV	CAGAAGGCGATGATAAGAAGTTCAA	
ECL_01761_L_CIV	CAGTAACCTCCACGTAGAAATCATC	PCR
ECL_01761_R_CIV	GATCAGGACGTCGATAAACTTATGG	
**Cluster IX**		
ECL_01758_L_CIX	TTTTGTGAAGCTCTCCATCATGAAG	PCR
ECL_01758_R_CIX	CCGAACATGGTCAGAATATTGATGG	
ECL_01760_L_CIX	TGCATATAATGGATGAGAAAGTCGC	PCR
ECL_01760_R_CIX	AGCAGCACAGTGAACGTAATAATG	
ECL_01761_L_CIX	GTAAGAAGTTTAAATTCCGTCGTCG	PCR
ECL_01761_R_CIX	GATTACGTCATCAAACCTTTCAACC	
ECL_01762_L_CIX	TATAATTGCCTTTATCAGAGACGCC	PCR
ECL_01762_R_CIX	TATACGCTGTATAAATCCAGAACGG	
**Cluster XII**		
ECL_01759_L_CXII	TATGCTGCTGATCATTGAATTCTGG	PCR & qRT-PCR
For all other primers pairs	cf. cluster XI	
hsp60-F	GGTAGAAGAAGGCGTGGTTGC	ECL cluster identification (**40**)
hsp60-R	ATGCATTCGGTGGTGATCATCAG	

Table S3: Deoxynucleotide primers used in this study.
